# Cortical neurons derived from human pluripotent stem cells lacking FMRP display altered spontaneous firing patterns

**DOI:** 10.1186/s13229-020-00351-4

**Published:** 2020-06-19

**Authors:** Shreya Das Sharma, Rakhi Pal, Bharath Kumar Reddy, Bhuvaneish T. Selvaraj, Nisha Raj, Krishna Kumar Samaga, Durga J. Srinivasan, Loren Ornelas, Dhruv Sareen, Matthew R. Livesey, Gary J. Bassell, Clive N. Svendsen, Peter C. Kind, Siddharthan Chandran, Sumantra Chattarji, David J. A. Wyllie

**Affiliations:** 1 Centre for Brain Development and Repair, Institute for Stem Cell Biology and Regenerative Medicine, Bangalore, 560065 India; 2 The University of Trans-Displinary Health Sciences and Technology, Bangalore, 560064 India; 3 Centre for Clinical Brain Sciences, University of Edinburgh, Chancellor’s Building, Edinburgh, EH16 4SB UK; 4 UK Dementia Research Institute at the University of Edinburgh, Edinburgh Medical School, Chancellor’s Building, Edinburgh, EH16 4SB UK; 5 Department of Cell Biology, Emory University School of Medicine, Atlanta, GA 30322 USA; 6 The Board of Governors Regenerative Medicine Institute and Department of Biomedical Sciences, Cedars-Sinai Medical Center, Los Angeles, CA 90048 USA; 7 iPSC Core, The David Janet Polak Foundation Stem Cell Core Laboratory, Cedars-Sinai Medical Center, Los Angeles, CA 90048 USA; 8 Cedars-Sinai Biomanufacturing Center, West Hollywood, CA 90069 USA; 9 Department of Biomedical Sciences, Cedars-Sinai Medical Center, Los Angeles, CA 90048 USA; 10 Centre for Discovery Brain Sciences, University of Edinburgh, Hugh Robson Building, Edinburgh, EH8 9XD UK; 11 Patrick Wild Centre, University of Edinburgh, Hugh Robson Building, Edinburgh, EH8 9XD UK; 12 Simons Initiative for the Developing Brain, University of Edinburgh, Hugh Robson Building, Edinburgh, EH8 9XD UK; 13 National Centre for Biological Sciences, Tata Institute for Fundamental Research, Bangalore, 560065 India

**Keywords:** Fragile X syndrome, Disease-modelling, Electrophysiology, Action potential

## Abstract

**Background:**

Fragile X syndrome (FXS), a neurodevelopmental disorder, is a leading monogenetic cause of intellectual disability and autism spectrum disorder. Notwithstanding the extensive studies using rodent and other pre-clinical models of FXS, which have provided detailed mechanistic insights into the pathophysiology of this disorder, it is only relatively recently that human stem cell-derived neurons have been employed as a model system to further our understanding of the pathophysiological events that may underlie FXS. Our study assesses the physiological properties of human pluripotent stem cell-derived cortical neurons lacking fragile X mental retardation protein (FMRP).

**Methods:**

Electrophysiological whole-cell voltage- and current-clamp recordings were performed on two control and three FXS patient lines of human cortical neurons derived from induced pluripotent stem cells. In addition, we also describe the properties of an isogenic pair of lines in one of which *FMR1* gene expression has been silenced.

**Results:**

Neurons lacking FMRP displayed bursts of spontaneous action potential firing that were more frequent but shorter in duration compared to those recorded from neurons expressing FMRP. Inhibition of large conductance Ca^2+^-activated K^+^ currents and the persistent Na^+^ current in control neurons phenocopies action potential bursting observed in neurons lacking FMRP, while in neurons lacking FMRP pharmacological potentiation of voltage-dependent Na^+^ channels phenocopies action potential bursting observed in control neurons. Notwithstanding the changes in spontaneous action potential firing, we did not observe any differences in the intrinsic properties of neurons in any of the lines examined. Moreover, we did not detect any differences in the properties of miniature excitatory postsynaptic currents in any of the lines.

**Conclusions:**

Pharmacological manipulations can alter the action potential burst profiles in both control and FMRP-null human cortical neurons, making them appear like their genetic counterpart. Our studies indicate that FMRP targets that have been found in rodent models of FXS are also potential targets in a human-based model system, and we suggest potential mechanisms by which activity is altered.

## Background

Fragile X syndrome (FXS) is a leading genetic cause of intellectual disability and one of the most common monogenic causes of autism spectrum disorder. FXS results from the loss of expression of FMRP, the protein produced from the *FMR1* gene. As the *FMR1* gene is located on the X chromosome, FXS occurs more in males (1:4000) than in females (1:6000–8000) [1, 2]. Individuals with FXS exhibit a variety of symptoms—learning disabilities, anxiety, unstable mood, attention deficit, hyperactivity, seizures and altered social behaviour [3–5]. Extensive mechanistic studies in rodent models of FXS have shown that FMRP is a mRNA translational repressor [6], and a key feature found in pre-clinical models of FXS is dysregulation of protein homeostasis [7]. Furthermore, many of the proteins that are targets of FMRP are found at central synapses and/or influence neuronal excitability [8]. Indeed, a distinguishing feature in rodent models of FXS is altered neuronal integration of excitatory and inhibitory inputs and concomitant aberrant network activity [9–14]. Indeed, perturbation of the neuronal circuits and networks in the early stages of brain development is likely to be responsible for many of the impairments exhibited by individuals with a range of neurodevelopmental disorders. However, it is not always the case that all neuronal populations show hyperexcitability; a recent study using mouse primary cortical neurons demonstrated that loss of FMRP did not affect the basal neuronal excitability [15] while a study using foetal rat visual cortex showed the *Fmr1* null neurons to be hypoexcitable [16]. Indeed, such results which may be taken by some to be conflicting but equally could simply illustrate the complexity of studying FXS pathophysiology in rodent models.

While the extensive studies using rodent and other pre-clinical models of FXS have provided detailed mechanistic insights into the pathophysiology of this disorder, it is only relatively recently that human stem cell-derived neurons have been employed as a model system to further our understanding of the pathophysiological events that may underlie FXS [17–19]. To date, there have been relatively few studies that have examined the effects of loss of expression of FMRP in human neurons in the context of cellular excitability and network function [20–23]. Despite the paucity of such studies, there are differences reported in whether human *FMR1* null neurons display hypo- [21] or hyper- [23] activity. Furthermore, it is imperative that we assess whether key cellular phenotypes, first described in animal models of FXS, are present in human-derived cells. Moreover, potential therapeutic interventions developed from such pre-clinical studies have, to date, not been successful in developing treatments, and as such, the need for functional assessment in a ‘human-based’ platform offers the opportunity to expand our understanding of dysregulated signalling. Thus, not only are such studies essential to identify any species differences and translational understanding of FXS, they can also provide a ‘human-based’ platform to assess potential therapeutics.

Given the large number of FMRP targets that are associated with synaptic function and the well-described synaptic and network properties that are dysregulated in pre-clinical models of FXS, our present study focuses on assessing the physiological properties of two control (CON1, CON2) and three FXS patient lines (FXS1, FXS2, FXS3) of human cortical neurons derived from induced pluripotent stem cells (iPSCs). In addition, to ensure that the differences we observe in FXS patient lines are due to the silencing of the *FMR1* gene and the absence of FMRP and not differences in the different genetic backgrounds or ‘environmental’ influences on the iPSC-derived neurons, we also describe the properties of an isogenic pair of lines [24], one of which has had the *FMR1* gene genetically deleted (*FMR1*^−/y^) to allow direct comparison with an otherwise genetically identical line (*FMR1*^+/y^). Using electrophysiological recordings, we report that there is altered spontaneous neuronal action potential burst firing activity in all lines that lack FMRP. This occurs in the absence of differences in either intrinsic or synaptic properties in each of the lines, whether they express FMRP or not. We demonstrate through pharmacological approaches how this spontaneous action potential firing can be manipulated in order to understand the mechanistic basis underlying the dysregulated activity exhibited in neurons lacking FMRP.

## Materials and methods

### Generation of primary rodent astrocytes

Primary rodent astrocytes were isolated from the cerebral cortex of E18.5 CD 1 mice as described elsewhere [25]. Briefly, cortices were dissected, enzymatically digested and mechanically dissociated. Astrocytes were plated on poly-d-lysine and laminin (Sigma)-coated plates in medium containing DMEM supplemented with 10% FBS (Thermo Fisher Scientific) and passaged twice prior to co-culturing with neurons on a 24-well plate. For co-cultures, human NPCs (30,000 cells/coverslip) were plated on to a layer of astrocytes and differentiated into neurons in a humidified incubator (5% CO2) at 37 °C for 8 weeks.

### hiPSC and hESC lines

SC176 (apparently healthy control male) and SC128 (fragile X syndrome male) fibroblasts and hereafter referred to as CON1 and FXS1 lines, respectively, were obtained from Dr. Philip H. Schwartz at Children’s Hospital of Orange County’s National Human Neural Stem Cell Resource (http://nhnscr.org/). Fibroblast lines were reprogrammed at the Laboratory for Translational Cell Biology at Emory University (Atlanta, GA) using established protocols and under institutional approval. Induced human pluripotent stem cells (hiPSCs) were generated by transducing control and FXS patient fibroblasts with Sendai virus from the Cytotune 2.0 Reprogramming Kit (Thermo Fisher Scientific, Waltham, MA) as per the manufacturer’s protocol. Briefly, early passage fibroblasts were cultured in fibroblast medium (10% ES-qualified FBS, 0.1 mM NEAA, 55 μM β-mercaptoethanol, high glucose DMEM with Glutamax) for 2 days. On day 0, fibroblasts were transduced with Sendai virus cocktail of KOS, hc-Myc and hKlf4. Cells were fed with fibroblast medium every other day for 1 week. On day 7, cells were passaged onto vitronectin (Thermo Fisher Scientific)-coated dishes at a density of 250,000 to 500,000 cells/well. Beginning on day 8, cells were fed every day, and colonies began to emerge within another 7–10 days. Individual hiPSC colonies were manually picked and transferred to a dish coated with either vitronectin or Matrigel (BD Biosciences, San Jose, CA) and expanded as clonal lines. hiPSCs were maintained on Matrigel-coated dishes and fed every day with complete mTesR1 medium (Stem Cell Technologies). hiPSCs were passaged every 5–7 days using ReLeSR (Stem Cell Technologies, Cambridge, MA). ND30625 (apparently healthy control male), GM07072 (fragile X syndrome male) and GM05848 (fragile X syndrome male) fibroblasts and hereafter referred to as CON2, FXS2 and FXS3 lines, respectively, were obtained from the Coriell Institute of Medical Research under their consent and privacy guidelines as described on their website (http://catalog.coriell.org/). hiPSC generation was carried out at Cedars-Sinai Medical Centre (Los Angeles, CA). Fibroblasts were re-programmed into non-integrating and virus-free hiPSC by nucleofecting episomal plasmids expressing the reprogramming factors OCT4, SOX2, KLF4, L-MYC, LIN28 and shRNA to TP53. All newly reprogrammed hiPSC lines were confirmed to have normal karyotypes and passed a battery of characterization tests for pluripotency including hiPSC Scorecard Assay, PluriTest and RT-qPCR to confirm endogenous expression of pluripotency and confirm the lack of exogenous gene expression. The reprogrammed iPSC lines were also authenticated by matching the identity to the parental fibroblast sample by short tandem repeat (STR) profiling and compared to parental tissue source (fibroblasts). For STR profiling, the iPSC lines were authenticated by contracting with IDEXX Laboratories, Inc., and use of their CellCheck 9 service. All hiPSC lines were regularly karyotyped (WiCell, Madison, WI) and characterized for expression of markers of pluripotency using immunofluorescence and RT-PCR. The human embryonic stem cell (hESC) line Shef 4, hereafter referred to as *FMR1*^+/y^, was obtained from the UK Stem Cell Bank [26]. The Shef 4 *FMR1* null line, hereafter referred to as *FMR1*^−/y^, was generated using CRISPR-Cas9 technology as described previously [24]. hiPSC and hESC colonies were cultured and propagated in xeno-free and feeder-free conditions using Essential 8 medium (Thermo Fisher Scientific) in 6-well plates (Nunc Nunclon delta surface, Thermo Fisher Scientific) coated with reduced growth-factor Matrigel (Corning Inc, New York, NY) at 37 °C, 5% CO_2_ in a humidified incubator. The colonies were cultured to 90% confluence and enzymatically passaged for further propagation and cryopreservation [27]. Each of the cell lines used in this study are listed in Table 1; all experiments were performed after obtaining statutory institutional ethical clearances.

**Table 1:** Details of cell lines used in this study

ID in manuscript	ID at source	Age (years)	Sex	Reprogrammed cell line name	Re-programming method	Starting cell type	G band karyotype
CON1	SC176	14	M	SC176	Sendai virus	Fibroblast	Normal
CON2	ND30625	76	M	CS25iCTR-18nxx	Episomal vectors	Fibroblast	Normal
FXS1	SC128	23	M	SC128	Sendai virus	Fibroblast	Normal
FXS2	GM07072	22	M	CS072iFXS-n4	Episomal vectors	Fibroblast	Normal
FXS3	GM05848	4	M	CS8488iFXS-n5	Episomal vectors	Fibroblast	Normal
*FMR1*^+/y^	Shef 4	ESC	M	NA	NA	-	Normal
*FMR1*^−/y^	Shef 4-FMR1 null	ESC	M	ESC- CRISPR Cas 9 edited	NA	-	Normal

### Generation and expansion of cortical NPCs and neural differentiation

Cortical NPCs were derived from each of the cell lines mentioned above as described previously. Similarly, cryopreservation, maintenance, characterization and expansion of cortical NPCs were carried out according to previously published protocol [27]. Human cortical neurons were differentiated from cortical neural precursor cells as described earlier [27]. Briefly, cNPCs were plated on 13-mm glass coverslips (VWR, Radnor, PA) coated with poly-l-ornithine, laminin, fibronectin (Sigma, St. Louis, MO) and reduced growth-factor Matrigel at 30,000 cells/coverslip in default medium, maintained at 3% O_2_, 5% CO_2_, 37 °C for 1 week (days 1–7) followed by default medium with forskolin (Tocris Bioscience, Bristol, UK) for further 2 weeks (days 8–21) and supplemented with BDNF and GDNF (R&D Systems, Minneapolis, MN) for 5 weeks (days 22–56). Immunostaining and electrophysiological characterization was performed at the end of 8 weeks.

### Western blot analysis

hiPSC and hESC pellets were lysed using lysis buffer (50 mM Tris-HCL, pH 7.4, 2 mM EDTA, 0.1% SDS, 1% Triton-X 100, 0.5% Na-deoxycholate, 150 mM NaCl, protease and phosphatase inhibitor cocktails), sonicated (10 cycles of 30 s on/30 s off) and centrifuged at 13,000 rpm for 10 min. The supernatant was collected and protein estimation performed using BCA assay kit (Thermo Fisher Scientific). Twenty micrograms/sample protein was loaded on a precast NuPAGE 4–12% Bis-Tris Protein gel (Thermo Fisher Scientific) followed by protein transfer to a PVDF membrane (GE Healthcare, Life science, Chicago, IL). The blot was blocked in 1:1 TBST (0.2 M Trizma base, 0.15 M NaCl, 0.1% Tween-20) and Odyssey blocking buffer (Li-COR Bioscience, Lincoln, NE) for 1 h at room temperature. The membrane was incubated with primary antibodies FMRP (1:1000, AbCam, Cambridge, UK) and β-actin (1:5000, Sigma), in blocking buffer for overnight at 4 ^°^C, washed with TBST and incubated in secondary antibodies with IRDye 680RD and IRDye 800CW (Li-COR Bioscience) respectively for 1 h at room temperature. Following further washing, the blot was dried and imaged using Li-COR Odyssey FC infrared system.

### Immunocytochemistry and imaging

hiPSCs, hESCs, NPCs and neurons were stained as per standard immunocytochemistry protocols. Briefly, cells were fixed with 4% PFA for 10 min followed by permeabilization for 5 min using 0.3% Triton X (USB Corporation, Cleveland, Ohio, OH). Cells were incubated in 3% BSA (Sigma) for 30 min to block non-specific binding of antibodies. Further, cells were incubated in primary antibody at room temperature and secondary antibody consecutively for 1 h each, mounted onto glass slides using Fluorsave (Sigma) and stored at 4 °C in the dark till imaging. Images were acquired using a confocal laser scanning microscope (Fluoview 3000 Olympus, Japan). hiPSC and ESC imaging was done using a × 40 (1.3 NA) oil immersion objective, and NPCs and co-culture images were captured using × 60 (1.4 NA) oil immersion objective with diode lasers 405 nm, 488 nm, 561 nm and 640 nm. All images were captured at 512 × 512 pixels per inch; Z step size was set at 0.5 μm with 1 airy unit of pinhole diameter.

### Electrophysiology

Electrophysiological recordings were made at room temperature (23–24 °C), and similar protocols to those described previously [27–29] were used. Coverslips, containing human cortical neurons that had been cultured for 8 weeks, were transferred to a recording chamber, and whole-cell current- or voltage-clamp recordings were made using a MultiClamp 700B amplifier (Molecular Devices, San Jose, CA). Cells were perfused with an external recording solution comprising of (in mM): NaCl 152, KCl 2.8, HEPES 10, CaCl_2_ 2, glucose 10, pH 7.3–7.4 (300–320 mOsm) with a flow rate of approximately 1.35 ml per minute.

For current-clamp, recording patch-pipettes, fabricated from thick-walled borosilicate glass, were filled with an internal recording solution containing (in mM): K-gluconate 155, MgCl_2_ 2, HEPES 10, Na-PiCreatine 10, Mg_2_-ATP 2 and Na_3_-GTP 0.3, pH 7.3 (280–290 mOsm), and had resistances of 3–4 MΩ. The liquid junction potential was calculated to be +14 mV (JPCalc, Clampex), and the values for membrane potential reported here do not take liquid junction potential into consideration. For the assessment of intrinsic membrane properties and recording of spontaneous network activity, current-clamp recordings were performed at potentials of -60 mV and -70 mV respectively, with bridge balance mode and pipette capacitance neutralized. The duration of action potential bursts was measured from the start of the first action potential to the time of the last action potential with bursts being defined as two or more action potentials occurring during a period of depolarization. Data were filtered at either 3 kHz or 10 kHz, for voltage-clamp and current-clamp recordings, respectively, and digitized at 20 kHz, via a Digidata 1550 (Molecular Devices, San Jose, CA). Stimulation protocols were generated using pClamp 10.5 software, and subsequent offline analysis was conducted using Clampfit 10.5 software.

For voltage-clamp, recording patch-pipettes were filled with an internal solution consisting of (in mM): Cs-gluconate 110, CsCl 20, HEPES 10, NaCl 4, QX-314 5, EGTA 0.2, Na-PiCreatine 10, Mg_2_-ATP 2 and Na_3_-GTP 0.3, pH 7.3 (280–290 mOsm), and neurons were clamped at −70 mV. Where recordings of miniature excitatory postsynaptic currents (mEPSCs) were performed, the external recording solution was supplemented with tetrodotoxin (TTX; 500 nM) and MgCl_2_ (1.3 mM) to block the NMDA receptor-component of synaptic events. To record the persistent sodium current (*I*_NaP_), neurons were clamped at −80 mV, then stepped to −100 mV, and a depolarizing voltage ramp [30] to −20 mV (20 mV/s) was applied. *I*_NaP_ was isolated by subtracting the current recorded in the presence of TTX (1 μM) from current recorded immediately prior in the same neuron but in the absence of TTX. To record the Ca^2+^-activated BK current (*I*_BKCa_), neurons were clamped at −60 mV, then stepped to −110 mV, and a depolarizing voltage ramp [31] to +40 mV (100 mV/s) was applied, with neurons being held at +40 mV for a further 50 ms. During these recordings, the external recording solution was supplemented with TTX (0.5 μM) and with *I*_BKCa_ being isolated by subtracting the current recorded in the presence of paxilline (10 μM) from current recorded immediately prior in the same neuron but in the absence of paxilline.

### Statistical analysis

All values are expressed as mean ± standard error of the mean (SEM), and each data set was assessed for normality. For the analysis of intrinsic membrane properties, mEPSC and network recordings, one-way repeated analysis of variance (ANOVA) followed by post hoc Tukey’s test was used. Paired statistical tests were used for data sets before and after drug application. Paired *t* test was used for data sets that passed the normality test, and Wilcoxon test was used for data sets that failed the normality test. Two-way repeated measures ANOVA, followed by post hoc Tukey’s test, was used for *I*_NaP_ and *I*_BKCa_ current–voltage relationships. All statistical tests were performed using GraphPad Prism (GraphPad software Inc., La Jolla, CA, RRID: SCR_002798). The number of experimental replicates (cells) is denoted as ‘*n*,’ while ‘*N*’ represents number of de novo preparations of batches from which ‘*n*’ is obtained.

## Results

### Absence of FMRP does not affect differentiation efficiency of FMRP-lacking neurons

**Fig. 1:**
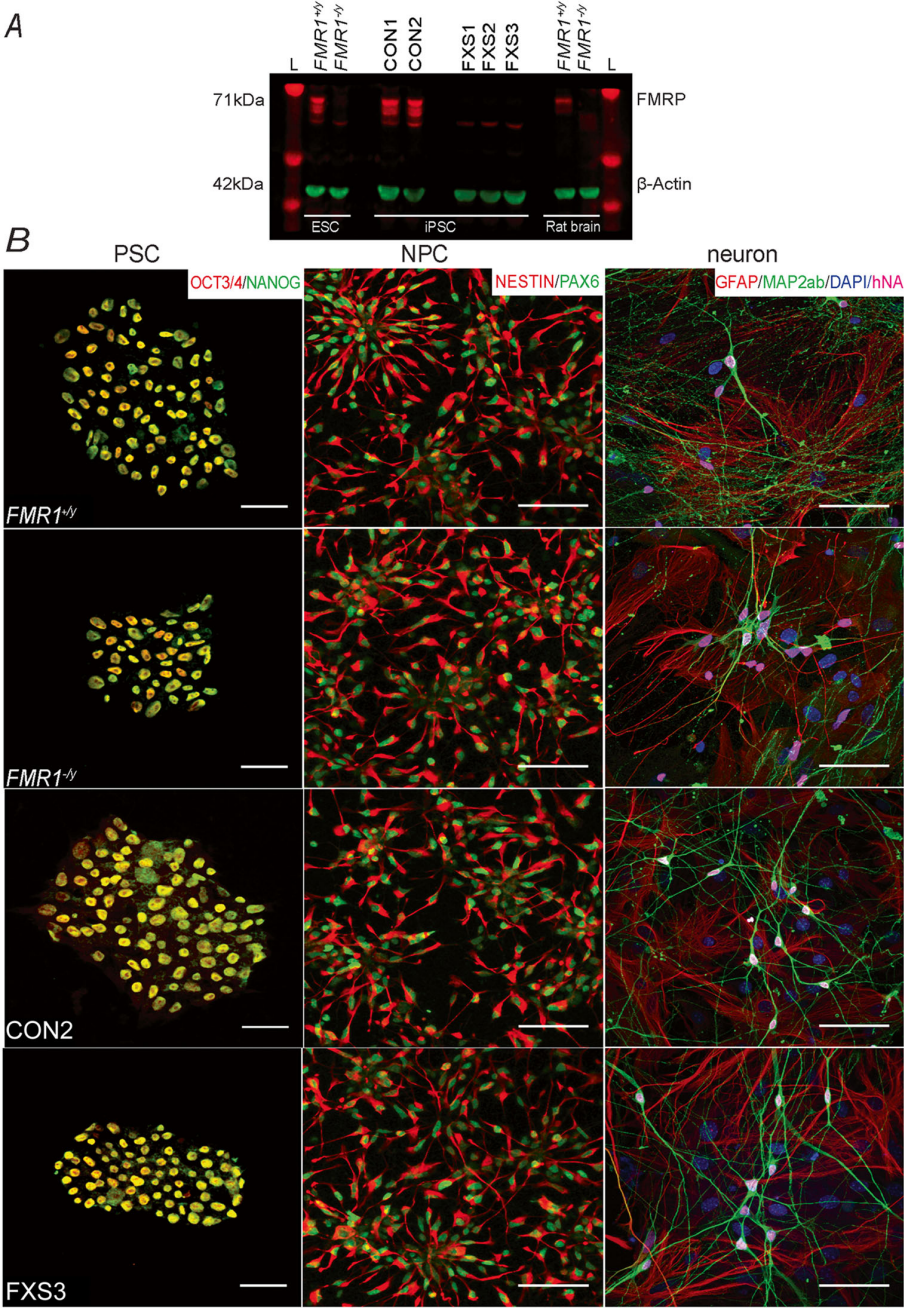
Specification of neurons from human stem cell and pluripotent stem cell-derived neural progenitor cells. **a** LI-COR immunoblot image of FMRP obtained from each of the pluripotent stem cell lines examined. The image illustrates that *FMR1*^*+/y*^, CON1 and CON2 cell lines express FMRP (71 kDa), and *FMR1*^−*/*y^, FXS1, FXS2 and FXS3 lines lack FMRP. Rat brain lysate from *Fmr*^*+/y*^ and *Fmr1*^−*/y*^ animals were used as positive and negative controls respectively. **b** Left panel: representative confocal images of paraformaldehyde fixed hESC (*FMR1*^*+/y*^ and *FMR1*^−*/y*^) and hiPSC (CON2 and FXS3) expressing the pluripotency markers, Nanog and Oct3/4. Scale bar = 50 μm. Middle panel: immunofluorescent staining of NPCs arranged as rosettes (derived from either hESC or hiPSC) for the progenitor markers Nestin and PAX6. Scale bar = 50 μm. Right panel: confocal images illustrating hESC- and hiPSC-derived cortical neurons expressing Map2ab/DAPI/hNA. The GFAP-positive cells are murine astrocytes (negative for human nuclei) with which hESC- and hiPSC-derived neurons were co-cultured. Scale bar = 50 μm. Abbreviations: hESC, human embryonic stem cells; hiPSC, human pluripotent stem cells; *FMR1*^*+/y*^, human embryonic stem cell line; *FMR1*^−*/y*^, gene-edited isogenic hESC pair lacking *FMR1* gene; L, Ladder; CON1, CON2, control hiPSC line; FXS1, FXS2 and FXS3, hiPSC lines from fragile X syndrome patients

Fibroblast-derived iPSCs were generated from two healthy individuals (CON1 and CON2), three FXS patients lacking FMRP (FXS1, FXS2 and FXS3) and one isogenic embryonic stem cell (*FMR1*^*+/y*^_;_*FMR1*^*−/y*^) pair where the *FMR1* gene was deleted using CRISPR/Cas9-mediated genome editing [24]. The absence of FMRP in FXS1, FXS2, FXS3 and *FMR1*^*−/y*^ PSC colonies was confirmed using western blot analysis as shown in Fig. 1a (*N* = 3). All cell lines expressed pluripotent stem cell markers (Oct3/4 and Nanog) as shown in Fig. 1b (left panel, *N* = 3; *n* = 9). NPCs were generated from PSC and further terminally differentiated into neurons using previously published protocols [27]. Immunocytochemistry showed high expression of NPC markers, Nestin and PAX6 across all samples (middle panel, *N* = 6; *n* = 18). Immuno-labelling of co-cultures at 8 weeks post differentiation revealed cells positive for Map2ab and human nuclear antigen (hNA) confirming all neurons to be of human origin only (Fig. 1b right panel, *N* = 6; *n* = 18). Comparable differentiation efficiency was observed across all 7 lines. These data demonstrate that loss of FMRP does not affect the differentiation potential of human PSC-derived cortical neurons in vitro.

### Human neurons lacking FMRP display altered spontaneous action potential burst firing patterns

**Fig. 2:**
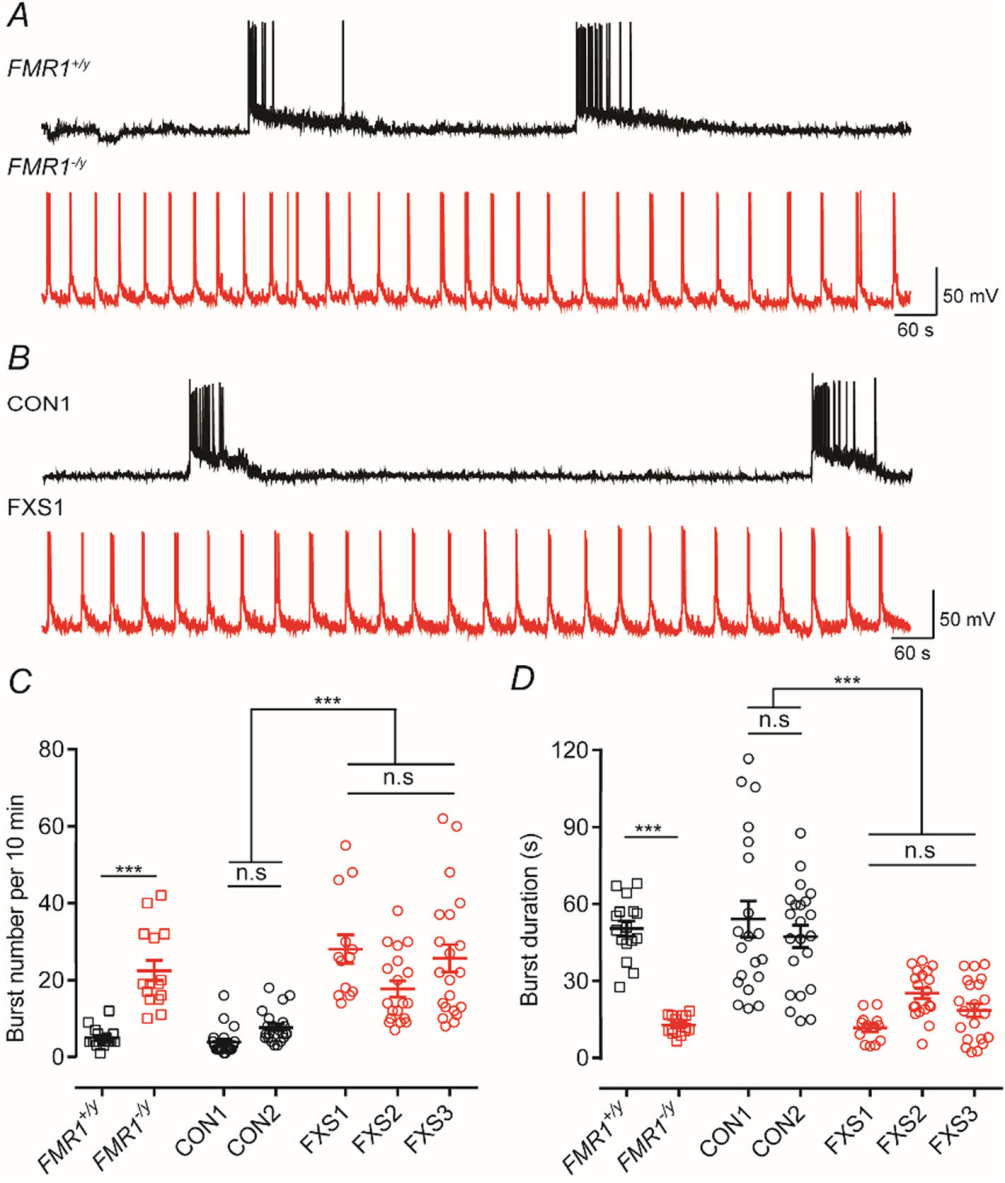
Bursts of action potentials occur at high frequencies but with shorter durations in neurons lacking FMRP. **a** Representative current-clamp recording (*V*_hold_ = −70 mV) of spontaneous bursts from either *FMR1*^*+/y*^ (black) or *FMR1*^−*/y*^ (red) neuron illustrating the difference in action potential bursting profiles. **b** As in **a** but illustration recordings from hiPSC lines CON1 (black) and FXS1 (red). **c** Mean burst number per 10 min of recording for each of the hESC and hiPSC lines. Overall, the mean numbers for bursts (per 10 min) are 5 ± 0.65 (*FMR1*^*+/y*^; *n* = 16, *N* = 3); 22.53 ± 2.63 (*FMR1*^−*/y*^; *n* = 15, *N* = 3); 3.9 ± 0.81 (CON1; *n* = 20, *N* = 3); 7.727 ± 0.88 (CON2; *n* = 22, *N* = 3); 28.07 ± 3.73 (FXS1; *n* = 13, *N* = 3); 17.74 ± 2.10 (FXS2; *n* = 19, *N* = 3); and 25.71 ± 3.56 (FXS3; *n* = 21, *N* = 3). **d** Mean burst duration for each of the hESC and hiPSC lines. Overall, the mean durations are 50.4228 ± 2.88 s (*FMR1*^*+/y*^); 12.85 ± 0.92 s (*FMR1*^−*/y*^); 54.15 ± 7.03 s (CON1); 47.38 ± 4.30 s (CON2); 11.66 ± 1.51 s (FXS1); 25.25 ± 2.1 s (FXS2); 18.55 ± 2.58 s (FXS3). ***p < 0.001, one-way ANOVA with post hoc Tukey’s test

Recordings from principal neurons from *Fmr1*^*−*/y^ mice have shown increased action potential bursting in the somatosensory cortex and hippocampus [12, 32]. Moreover, in vitro cultures of cortical neurons derived from human pluripotent stem cells develop synchronous burst activity [33, 34]. Thus, to assess the effects of the loss of FMRP on the network bursting, we examined spontaneous (action potential-driven) activity in both the isogenic hESC lines and the hiPSC lines. Figure 2a, b illustrates that in neurons lacking FMRP current-clamp recordings show that the nature of bursts of action potentials is considerably different to those seen in neurons where FMRP is present. In *FMR1*^+/y^, CON1 and CON2 line bursts of action potentials occur at a low frequency (Fig. 2c) but have long durations (Fig. 2d) as defined by the interval between the first and last action potential in a burst. In contrast, those lines lacking FMRP (*FMR1*^−/y^, FXS1, FXS2 and FXS3) gave rise to bursts that occurred at greater frequencies but with shorter durations. While not quantified in terms of their action potential bursting patterns, evidence for increased frequencies of firing in iPSC-derived neurons from FXS patients has recently been reported [23].

**Fig. 3:**
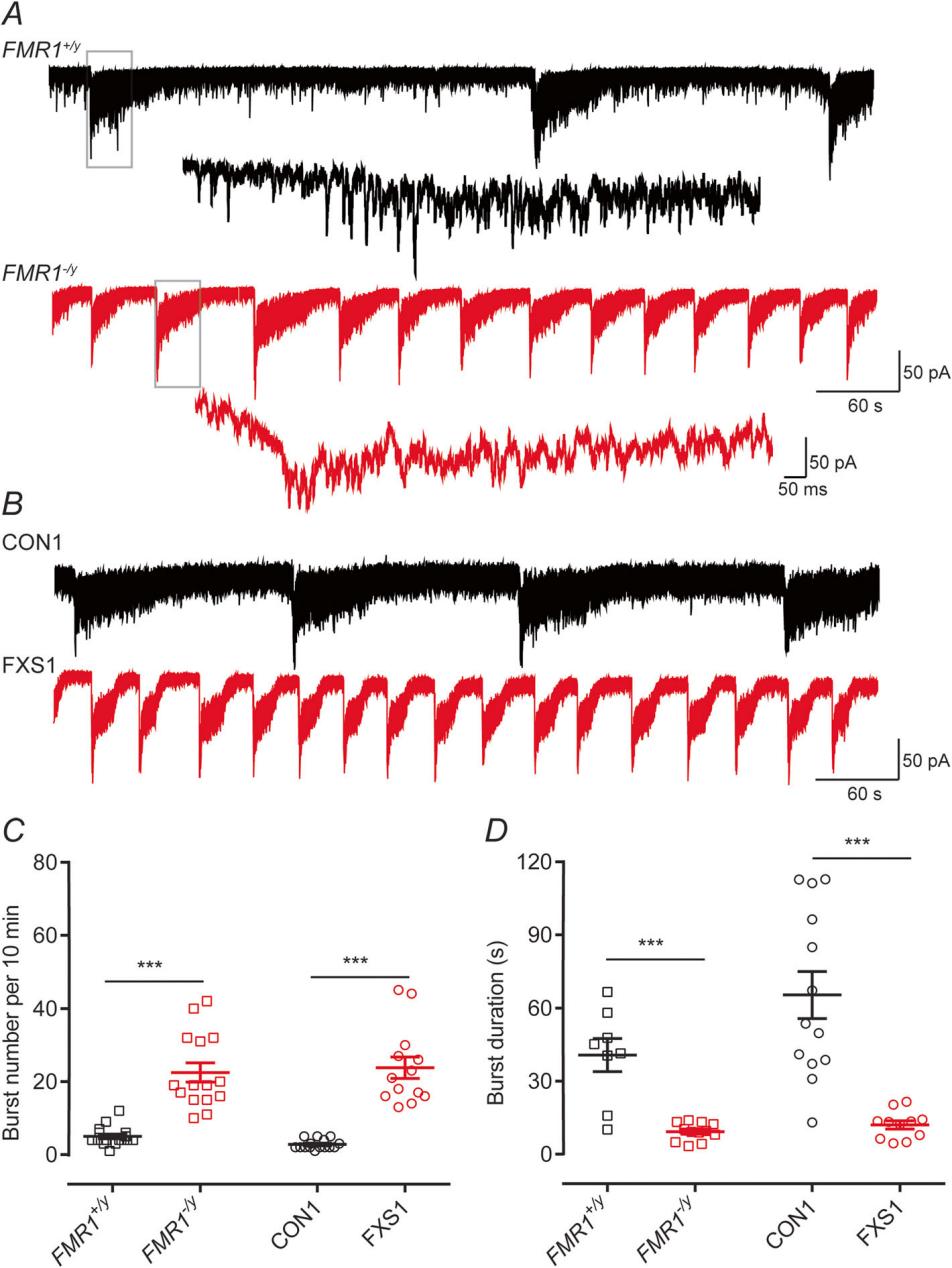
Neurons lacking FMRP display bouts of inward current activity of higher frequencies but of shorter durations compared to control neurons. **a** Representative voltage-clamp recording (V_hold_ = −70 mV) of spontaneous inward currents from either *FMR1*^+/y^ (black) or *FMR1*^−/y^ (red) neurons illustrating the difference in spontaneous events. The insets below, each trace illustrates an expanded time-base where evidence of synaptic currents underlying these inward currents can be observed. **b** As in **a** but illustration recordings from hiPSC lines CON1 (black) and FXS1 (red). **c** The number of events is higher in neurons lacking FMR1 than control. Overall, the mean number for bursts (per 10 min) are 4.5 ± 0.56 (*FMR1*^*+/y*^; *n* = 8, *N* = 3); 20.67 ± 3.96 (*FMR1*^−*/y*^; *n* = 12, *N* = 3); 2.8 ± 0.34 (CON1; *n* = 13, *N* = 3); 23.85 ± 2.92 (FXS1; *n* = 11, *N* = 3). **d** Mean burst durations recorded from neurons lacking FMRP are shorter than those from control lines. Overall, the mean durations are 40.72 ± 6.8 s (*FMR1*^*+/y*^); 9.28 ± 1.05 s (*FMR1*^−*/y*^); 59.90 ± 9.07 s (CON1); 11.52 ± 1.55 s (FXS1). ***p < 0.001, paired *t* test, Wilcoxon test

In voltage-clamp, the network activity occurring in a culture could readily be identified as large inward currents that lasted for several seconds and which occurred at regular frequencies (Fig. 3). As with the current-clamp recordings, the duration of these inward currents were longer and occurred at lower frequencies for neurons derived from *FMR1*^+/y^ and CON1 lines compared to those from *FMR1*^*−*/y^ and FXS1 lines (Fig. 3a, b). Bursting activity was NMDA-receptor dependent as it was abolished by applying AP-5 to the external recording solution (data not shown) and as indicated by the expanded traces (Fig. 3a) resulting from the summation of many individual synaptic events impinging on a neuron simultaneously. In the current-clamp recordings (Fig. 2), the synaptic events drive the waves of depolarization and result in the trains of action potentials that are observed. Quantification of the number and duration of these events (Fig. 3c, d) revealed statistically significant differences in nature of the activity seen in *FMR1*^+/y^, CON1 and CON2 lines compared with those lines that lacked expression of FMRP with the duration of the voltage-clamp recorded events matching those recorded in current-clamp. The presence of wild-type cortical mouse astrocytes has been shown to rescue morphological differences observed in *Fmr1*^*−*/y^ mouse hippocampal neurons [35]. However, the distinct neuronal firing profiles we report are obtained when human neurons expressing or lacking FMRP are co-cultured with wild-type (mouse) astrocytes. This indicates that using the culture conditions employed in this study, these action potential bursting phenotypes are cell-autonomous. We next turned our investigation to assess whether the observed differences were due to either intrinsic or synaptic properties of neurons lacking FMRP.

### Despite different spontaneous action potential firing patterns, absence of FMRP in human cortical neurons does not alter intrinsic or synaptic properties

Given the distinct patterns of spontaneous burst firing in neurons lacking FMRP compared to control neurons, we sought to determine whether this was due to differences in either intrinsic or synaptic properties between cell lines. Indeed, rodent models of FXS have reported that some classes of principal neurons lacking FMRP display altered intrinsic excitability [9, 12–14, 36] although others have not observed difference in these parameters [15, 37] most likely reflecting the fact that where differences do occur these are cell-type specific, rather than a generalized alteration in intrinsic excitability due to the loss of expression of FMRP. However, human neurons lacking FMRP have been reported to exhibit a considerably compromised ability to generate trains of action potential firing in response to depolarizing current injections [21]. However, using the protocols described above to generate human excitatory cortical neurons, we observed no differences in either passive or active membrane properties in any of the lines we studied (Fig. S1). Specifically, our data do not align with this previous study [21] that reported that human ESC-derived neurons lacking FMRP were only capable of firing, at most, one action potential in response to depolarising current injections.

To assess whether, with our protocols, loss of FMRP alters synaptic activity, we recorded the mEPSCs from hESC- and hiPSC-derived neurons. Previous studies of hiPSC-derived cortical neurons have suggested that neurons lacking FMRP possess low levels of spontaneous synaptic activity [20, 21]. Notwithstanding, the spontaneous synaptic activity as assessed by recording mEPSCs (Fig. S2A, B) indicated that mEPSC frequencies (Fig. S2C) and their amplitudes (Fig. S2D) were not different in neurons lacking FMRP compared to control.

While we could not find differences in either intrinsic or synaptic properties between cells that either expressed FMRP or lacked FMRP, the fundamentally different firing patterns indicate that there must be a differential regulation of some ionic conductances that contribute to the bursting profiles. Thus, we next considered whether, through pharmacological manipulations, we could alter the nature of the bursting activity observed in each of the sets of cell lines.

### Neurons lacking FMRP show reduced *I*_NaP_ densities and their bursting properties are insensitive to riluzole

**Fig. 4:**
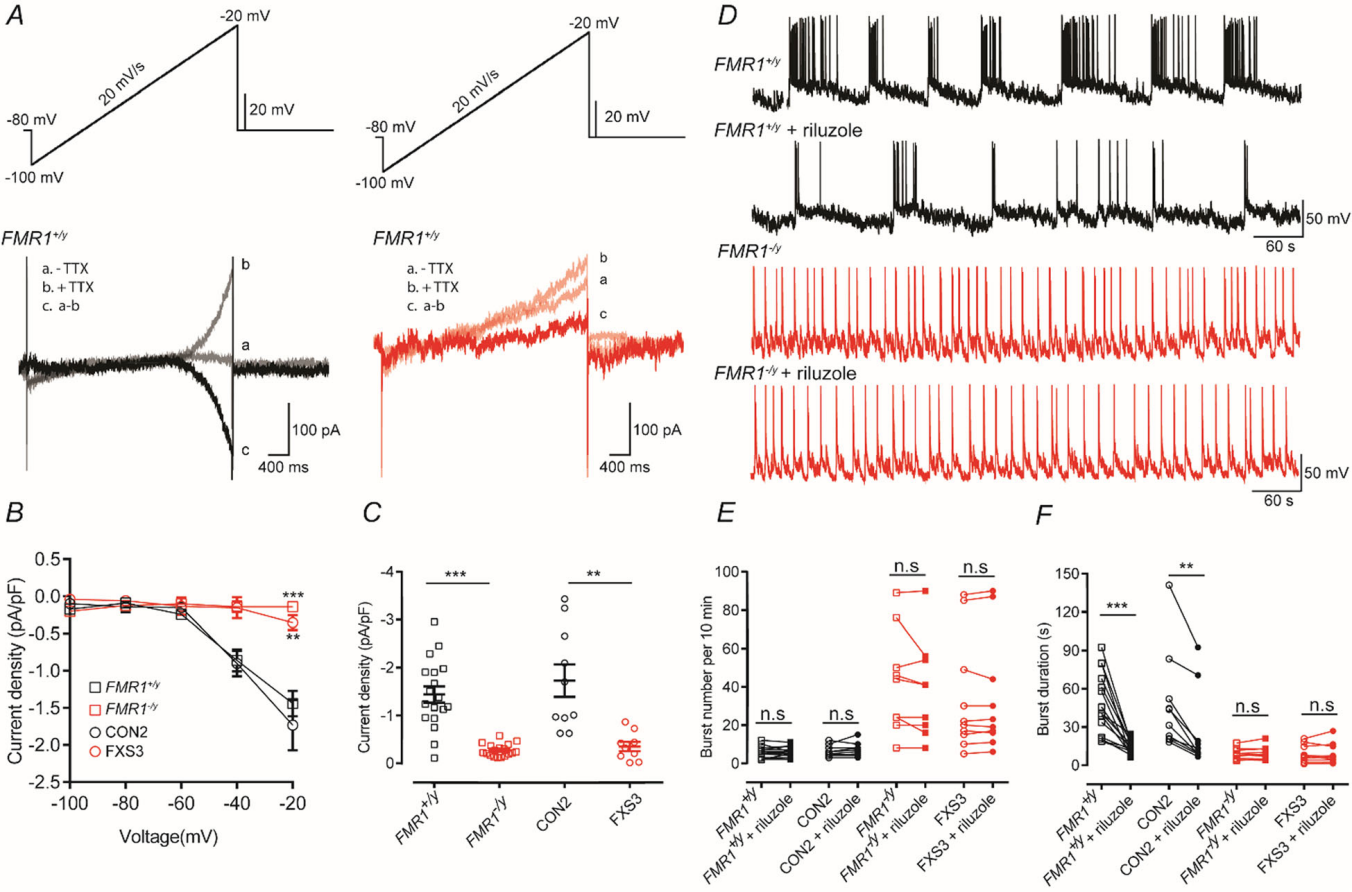
Neurons lacking FMRP have reduced *I*_NaP_ densities, and their burst properties are insensitive to riluzole. **A** Persistent sodium currents (*I*_NaP_) evoked by a slow depolarizing ramp (−100 to −20 mV, 20 mV/s) either before (*traces* a) or during (*traces* b) application of TTX. The TTX-sensitive current (*traces* c) was isolated by subtracting *traces* a and b. **B** I–V curves plotted from the ramp-evoked *I*_NaP_, showing the decreased *I*_NaP_ in neurons lacking FMRP. Currents are normalized to the corresponding cell capacitance. **C** Quantification of current density at −20 mV show significant decrease of the persistent sodium current in neurons lacking FMRP. Overall, the mean current densities are −1.44 ± 0.17 (*FMR1*^*+/y*^, *n* = 18, *N* = 3); −1.73 ± 0.33 (CON2, *n* = 10, *N* = 3); −0.27 ± 0.03 (*FMR1*^−*/y*^, *n* = 18, *N* = 3); −0.35 ± 0.098 (FXS3, *n* = 9, *N* = 3). **p < 0.01, ***p < 0.001, two-way repeated measures ANOVA with post hoc Tukey’s test, paired *t* test and Wilcoxon test. **D** Representative traces illustrating spontaneous bursts recorded from either a *FMR1*^*+/y*^ neuron or a *FMR1*^−*/y*^ neuron before and after the application of riluzole (0.1 μM). **E** Quantification of the number of bursts recorded from *FMR1*^*+/y*^, CON2, *FMR1*^−*/y*^, or FXS3 neurons before (open symbols) or after (closed symbols) application of riluzole. Overall, the mean number of bursts recorded are 5.733 ± 0.72 (*FMR1*^*+/y*^, *n* = 15, *N* = 3); 5.733 ± 0.65 (*FMR1*^*+/y*^ + riluzole, *n* = 15, *N* = 3); 6.3 ± 0.9 (CON2, *n* = 10, *N* = 3); 7 ± 1.12 (CON2 + riluzole, *n* = 10, *N* = 3); 42.33 ± 8.92 (*FMR1*^−*/y*^, *n* = 9, *N* = 3); 38.89 ± 8.49 (*FMR1*^*−/y*^ + riluzole, *n* = 9, *N* = 3); 34.1 ± 9.52 (FXS3, *n* = 10, *N* = 3); 34.3 ± 9.62 (FXS3 + riluzole, *n* = 10, *N* = 3). **F** Quantification of the duration of bursts recorded from *FMR1*^*+/y*^ , CON2, *FMR1*^−*/y*^, or FXS3 neurons before (open symbols) or after (closed symbols) application of riluzole. Overall, the mean burst durations are 47.86 ± 6.27 s (*FMR1*^*+/y*^); 14.14 ± 1.33 s (*FMR1*^*+/y*^ + riluzole); 47.81 ± 12.14 s (CON2); 25.55 ± 9.54 s (CON2 + riluzole); 8.77 ± 1.34 s (*FMR1*^−*/y*^ ); 9.43 ± 1.62 s (*FMR1*^*−/y*^ + riluzole); 8.91 ± 2.19 s (FXS3); 9.01 ± 2.52 (FXS3 + riluzole) **p < 0.01; ***p < 0.001, paired *t* test, Wilcoxon test

Recent studies have suggested that the altered excitability of neurons lacking FMRP in a mouse model of FXS is due to altered persistent sodium current (*I*_NaP_) activity [30, 38]. Although as indicated in Figure S1 we do not observe different action potential firing in response to relatively brief direct depolarizing current injection in each of the cell lines examined, it is likely that during prolonged periods of depolarization altered properties of intrinsic membrane currents underlie the different types of activity we observe. Indeed, *I*_NaP_ is intimately associated with determining the properties of burst firing of neurons [39–43]. A slow depolarizing voltage ramp was applied to neurons and the *I*_NaP_ pharmacologically isolated by recording in the absence and presence of TTX [30, 44]. Figure 4a illustrates persistent sodium current recordings from *FMR1*^+/y^ and *FMR1*^*−*/y^ neurons. The magnitude of *I*_NaP_ in *FMR1*^*−*/y^ and FXS3 lines is significantly reduced compared to those in *FMR1*^+/y^ and CON2 lines. These significant decreases in *I*_NaP_ are quantified in Fig. 4b, c. As illustrated in Fig. 4a–c the magnitude of *I*_NaP_ was significantly greater in *FMR1*^+/y^ and CON2 lines than that of either *FMR1*^*−*/y^ or FXS3 lines.

Given the significantly lower magnitude of *I*_NaP_ in neurons lacking FMRP, we hypothesized that pharmacological block of *I*_NaP_ would alter the burst parameters of control neurons such that they would resemble the neurons lacking FMRP. As illustrated and quantified in Fig. 4d–f the *I*_NaP_ blocker, riluzole (0.1 μM), significantly reduced the duration of action potential bursts in each of the *FMR1*^+/y^ and CON2 lines without affecting their overall frequency, thereby confirming the role of *I*_NaP_ in determining the action potential burst profile in human cortical neurons. Moreover, riluzole had no effect on the action potential burst parameters in *FMR1*^−/y^ and FXS3 neurons as might be expected given the low density of *I*_NaP_ in these cells. These data are in contrast with studies using *Fmr1*^−/y^ mice where it has been reported that *I*_NaP_ is enhanced in neurons lacking FMRP [30, 38].

### In neurons lacking FMRP, veratridine decreases action potential burst frequencies but increases their duration

**Fig. 5:**
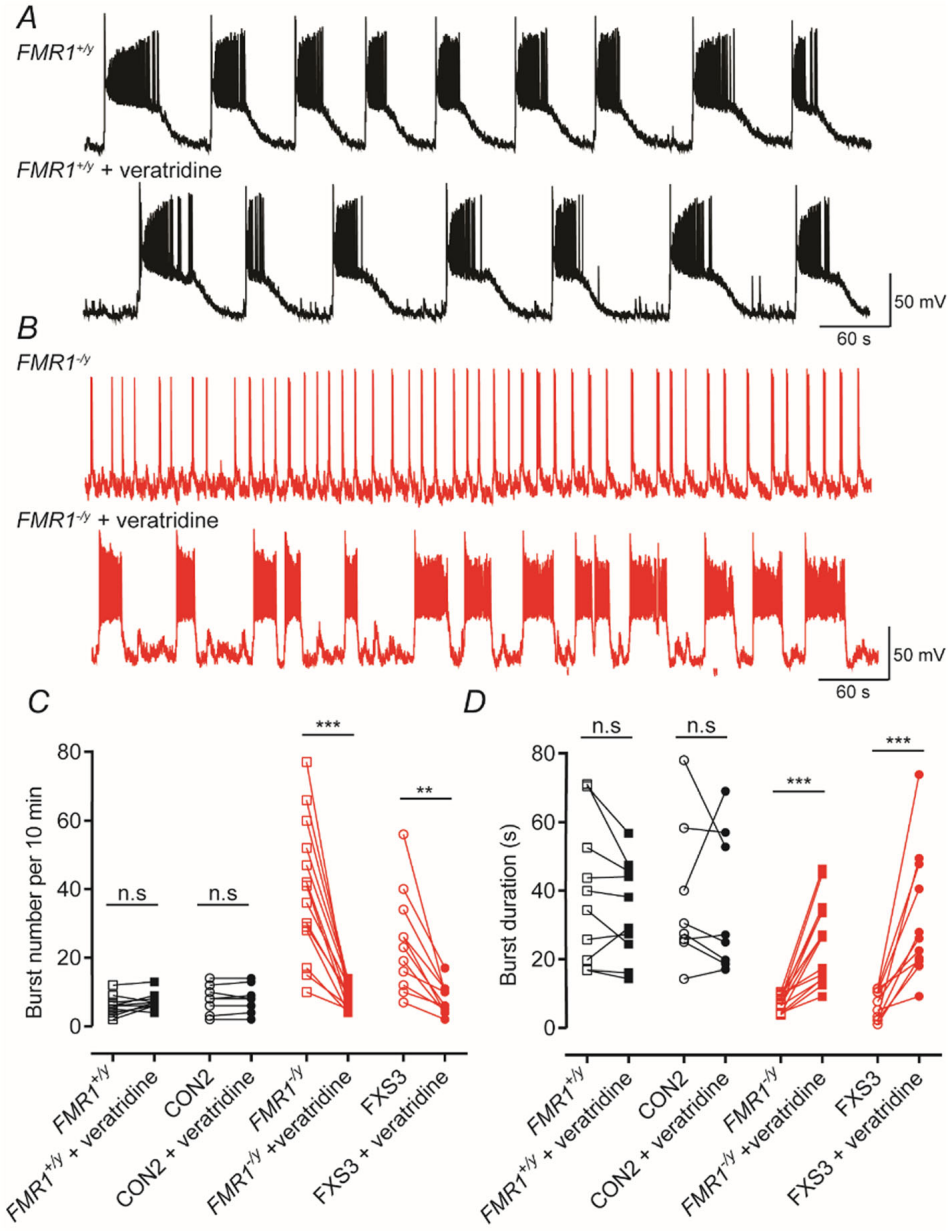
In neurons lacking FMRP activation of voltage-dependent Na^+^ channels decreases action potential burst frequencies but increases their duration. **a** Representative traces of a *FMR1*^*+/y*^ neuron illustrating spontaneous action potential bursting before and after the application of veratridine (0.5 μM). **b** As in **a** but illustrating traces obtained from a *FMR1*^−*/y*^ neuron. **c** Quantification of burst number in *FMR1*^+/y^, CON2, *FMR1*^−/y^ and FXS3 lines before and after the application of veratridine. Veratridine reduced significantly the frequency of bursts in lines lacking FMRP. Overall, the mean number of bursts are 6 ± 0.92 (*FMR1*^*+/y*^, *n* = 10, *N* = 3); 7.3 ± 0.76 (*FMR1*^*+/y*^ + veratridine, *n* = 10, *N* = 3); 7.87 ± 1.46 (CON2, *n* = 8, *N* = 3); 8 ± 1.45 (CON2 + veratridine, *n* = 8, *N* = 3); 39.29 ± 5.27 (*FMR1*^−*/y*^ , *n* = 14, *N* = 3); 8.857 ± 0.98 (*FMR1*^*−/y*^ + veratridine, *n* = 14, *N* = 3); 24.45 ± 4.37 (FXS3, *n* = 11, *N* = 3); 7.818 ± 1.32 (FXS3 + veratridine, *n* = 11, *N* = 3). **d** Quantification of action potential burst durations in each of the four lines examined. Veratridine increased significantly the duration of bursts in *FMR1*^−/y^ and FXS3 lines and had no overall effect on *FMR1*^+/y^ and CON2 lines. Overall, the mean burst durations are 39.09 ± 6.47 s (*FMR1*^*+/y*^); 34.34 ± 4.5 s (*FMR1*^*+/y*^ + veratridine); 37.42 ± 7.39 s (CON2); 35.77 ± 7.25 s (CON2 + veratridine); 7.318 ± 0.64 s (*FMR1*^−*/y*^ ); 25.5 ± 3.23 s (*FMR1*^*−/y*^ + veratridine); 7.004 ± 1.28 s (FXS3); 32.26 ± 5.66 (FXS3 + veratridine). For both **c** and **d**, **p < 0.01, ***p < 0.001, paired *t* test, Wilcoxon test

As illustrated above in the *FMR1*^−/y^ and FXS3 lines, the density of *I*_NaP_ was considerably reduced compared to that seen in *FMR1*^+/y^ and CON2 lines. Since *I*_NaP_ block does alter network properties in FMRP-containing neurons to resemble the bursting profile of FMRP-lacking neurons, we hypothesized that activation of voltage-gated Na^+^ channels would be able to correct the activity differences seen in FMRP null neurons. Indeed, the voltage-dependent Na^+^ channel activator, veratridine (0.5 μM), has a selective action on the burst firing patterns we recorded. As illustrated in Fig. 5, it had no significant effect on the burst parameters we measured in *FMR1*^+/y^ and CON2 lines but altered the bursting profile in *FMR1*^−/y^ and FXS3 lines (Fig. 5a, b). Veratridine significantly reduced the frequency of action potential bursts and increased the durations of *FMR1*^−/y^ and FXS3 lines (Fig. 5c, d). Thus, to some extent, activating voltage-dependent Na^+^ channels in neurons lacking FMRP gave rise to activity that phenocopies that seen in *FMR1*^+/y^ and CON2 lines.

### Neurons lacking FMRP show reduced *I*_BKCa_ densities, and their bursting properties are insensitive to paxilline

**Fig. 6:**
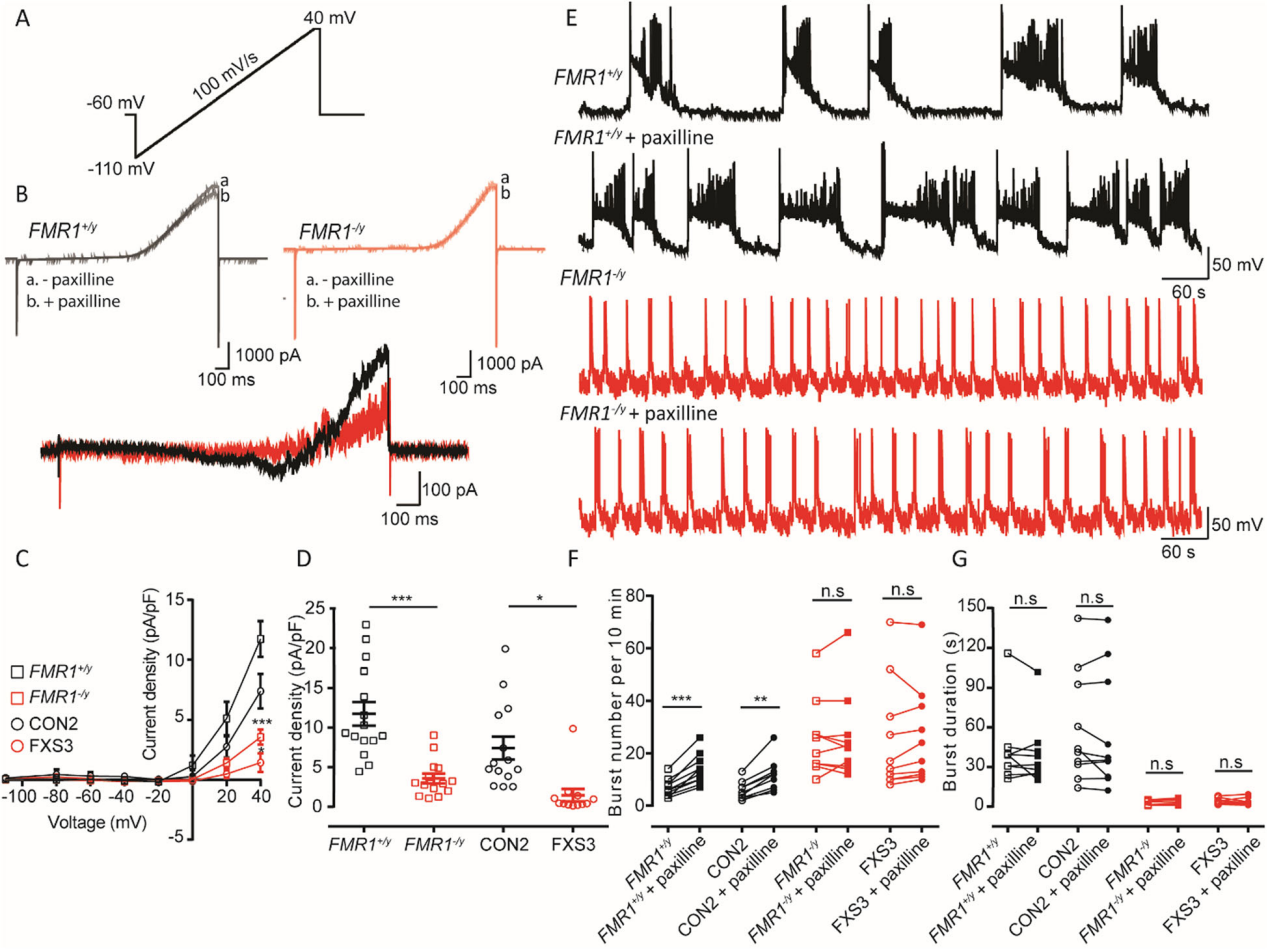
Neurons lacking FMRP have reduced *I*_BKCa_ densities and their burst properties are insensitive to paxilline. **a** Large Ca-activated potassium currents (*I*_BKCa_) evoked by a depolarizing voltage ramp from −110 to +40 mV, 10 mV/s, with a hold of 50 ms at +40 mV. **b** The *I*_BKCa_ was isolated by subtracting the currents recorded in the absence and presence of paxilline (10 μM). **c** I–V curves plotted from the ramp-evoked *I*_BKCa_ showing the decreased *I*_BKCa_ in neurons lacking FMRP. Currents are normalized to the corresponding cell capacitance. **d** Quantification of current densities at + 40 mV show significant decrease of *I*_BKCa_ in neurons lacking FMRP. Overall, the mean current densities are 11.73 ± 1.49 (*FMR1*^*+/y*^, *n* = 15, *N* = 3); 7.4 ± 1.43 (CON2, *n* = 14, *N* = 3); 3.57 ± 0.62 (*FMR1*^−*/y*^, *n* = 14, *N* = 3); 1.44 ± 0.78 (FXS3, *n* = 12, *N* = 3). *p < 0.05, ***p < 0.001, two-way repeated measures ANOVA with post hoc Tukey’s test, paired *t* test, Wilcoxon test. **e** Representative traces illustrating spontaneous bursts recorded from either a *FMR1*^*+/y*^ neuron or a *FMR1*^−*/y*^ neuron before and after the application of paxilline (10 μM) and illustrating the increase of action potential burst frequencies in *FMR1*^+/y^ neurons in the presence of paxilline. **f** Quantification of the number of bursts recorded from *FMR1*^*+/y*^, CON2, *FMR1*^−*/y*^, or FXS3 neurons before (open symbols) or after (closed symbols) application of paxilline. Overall, the mean number of bursts are 6.7 ± 1.086 (*FMR1*^*+/y*^, *n* = 10, *N* = 3); 13.8 ± 1.85 (*FMR1*^*+/y*^ + paxilline, *n* = 10, *N* = 3); 5.6 ± 1.08 (CON2, *n* = 10, *N* = 3); 11.3 ± 1.94 (CON2 + paxilline, *n* = 10, *N* = 3); 25.5 ± 4.51 (*FMR1*^−*/y*^ , *n* = 10, *N* = 3); 26 ± 5.12 (*FMR1*^*−/y*^ + paxilline, *n* = 10, *N* = 3); 25.4 ± 6.6 (FXS3, *n* = 10, *N* = 3); 26.5 ± 5.95 (FXS3 + paxilline, *n* = 10, *N* = 3). **g** Quantification of the duration of bursts recorded from *FMR1*^*+/y*^, CON2, *FMR1*^−*/y*^, or FXS3 neurons before (open symbols) or after (closed symbols) application of paxilline. Overall, mean burst duration are 40.97 ± 8.68 s (*FMR1*^*+/y*^); 38.47 ± 7.85 s (*FMR1*^*+/y*^ + paxilline); 58.65 ± 13.12 s (CON2); 56.08 ± 14.04 s (CON2 + paxilline); 2.65 ± 0.53 s (*FMR1*^−*/y*^); 3.08 ± 0.68 s (*FMR1*^*−/y*^ + paxilline); 4.107 ± 0.64 s (FXS3); 3.93 ± 0.77 (FXS3 + paxilline). **p < 0.01, ***p < 0.001, paired *t* test, Wilcoxon test

A variety of mRNAs encoding potassium channel subunits are targets for FMRP [8, 45]. Moreover, FMRP interacts with the auxiliary β4 subunit of the calcium-activated potassium channel (BK_Ca_) leading to increased activation of this current [31]. Indeed in studies of *Fmr1*^−/y^ mice, the reduced activity of the BK_Ca_ current (*I*_BKCa_) in the pyramidal neurons of hippocampus and cortex can be ameliorated by the genetic over-expression of the β4 subunit [32] or by the administration of a BK_Ca_ channel activator [46]. Thus, we wanted to determine whether in human neurons lacking FMRP there was similarly a reduced density of *I*_BKCa_. A depolarizing voltage ramp [32] was applied to neurons, and the *I*_BKCa_ was pharmacologically isolated by recording in the absence and presence of a specific BK_Ca_ channel blocker, paxilline (Fig. 6a, b). As illustrated in Fig. 6c, d and consistent with previous studies in mice [31], the magnitude of *I*_BKCa_ was significantly reduced in *FMR1*^−/y^ and FXS3 lines as compared to either *FMR1*^+/y^ or CON2 lines. Again, given the difference in magnitude of this current in lines expressing FMRP and those lacking FMRP, we would expect that blocking *I*_BKCa_ would alter the action potential burst profile of *FMR1*^+/y^ and CON2 neurons to resemble *FMR1*^−/y^ or FXS3 neurons. As illustrated in Fig. 6e, f, we confirmed this by showing that paxilline (10 μM) increased the number of action potential bursts occurring in a 10-min recording period in *FMR1*^+/y^ and CON2 neurons but was without effect in *FMR1*^−/y^ or FXS3 neurons. Nevertheless, and as quantified in Fig. 6g, paxilline did not alter, significantly, the duration of the action potential bursts in *FMR1*^+/y^ and CON2 neurons (or indeed in *FMR1*^−/y^ or FXS3 neurons). These results suggest that BK_Ca_ channels play a critical role in determining action potential burst frequencies in these neurons. Thus, as described above, we are able to manipulate selectively the firing pattern in a manner that is dependent on FMRP expression.

## Discussion

Descriptions of altered cortical network excitability in FXS patients have been described (reviewed in [11]); however, the physiological detail underpinning these observations remains to be explored in a human context. Indeed, current mechanistic understanding of altered excitability is largely based on the description of altered network excitability in rodent-based FXS models. Moreover, while we and others exploit the advantages of such models to understand pathophysiology and dysfunction, we must also consider that they may not always reflect human-specific physiology. The temporal profile of FMRP express during the process of embryonic development is a critical factor in FXS. Studies have shown that despite CGG-repeat expansion, FMRP can be detected in the human embryonic tissue, until the end of the first trimester [47, 48]. This is not seen in genetically engineered rodent models [49, 50] indicating that such models might not recapitulate fully the aetiology of FXS in humans. Thus, the ability to model neurodevelopmental diseases using human pluripotent stem cells gives us the opportunity to extend our physiological understanding of FXS in a human context.

For the first time, this study provides mechanistic insight into how loss of FMRP can lead to aberrant network activity in human cortical neurons derived from pluripotent stem cells. Three key findings emerge from our study. First, human cortical neurons lacking FMRP displayed spontaneous burst firing of action potentials at a greater frequency but with shorter durations compared to human cortical neurons. Second, despite differences in spontaneous firing patterns, we could find no differences in the passive and active membrane properties of human cortical neurons lacking FMRP compared to those expressing FMRP. Finally, synaptic activity as assessed by measuring mEPSC amplitudes and frequencies showed no differences between FMRP-lacking and FMRP-expressing neurons. Critically, the difference in burst firing can be attributed, unequivocally, to the loss of FMRP expression as the firing properties of both iPSC FXS patient-derived cortical neurons were indistinguishable from those recorded in the *FMR1*^−/y^ line, a CRISPR-Cas9 gene-edited ESC line. Furthermore, each of the lines expressing FMRP (*FMR1*^+/y^, CON1 and CON2) also displayed firing properties (less frequent but longer bursts) that were indistinguishable from each other. The most parsimonious explanation for these findings is that the loss of expression of FMRP is responsible for the differences in the burst firing and not other environmental causes or impacts of genetic diversity between non-isogenic pairs.

The duration of the bursting activity we observed is determined by the temporal nature of the synaptic input received by any individual neuron. This can be concluded as voltage-clamp recordings indicated the duration of the synaptic current activity matches that of the action potential bursting activity observed in our current-clamp recordings. In other words, it is not the case, for example, that a similar duration period of synaptic activity triggers different duration bursting activity in control and FMRP-lacking neurons. Indeed, given the similarities in the active membrane properties of control and FMRP-lacking neurons, it is perhaps unlikely that ‘inputs’ of equivalent duration would result in different duration ‘outputs’. Of course, it is a matter of semantics as to whether it is the duration of bursting that drives the synaptic event duration or the duration of the synaptic event that drives the membrane depolarization and action potential bursting event.

Spontaneous network properties are influenced by active and passive membrane properties, and indeed, many studies have reported that in rodent models the loss of expression of FMRP leads to cortical hyperexcitability [9, 12–14, 36]. It is becoming increasingly clear that FXS pathophysiology as assessed in rodent models shows complex diversity. For example, a recent study using mouse cortical primary neurons from *Fmr1*^−/y^ mice has reported no change in intrinsic and synaptic excitability [15], while hypoexcitability has been reported in *Fmr1*^−/y^ neurons from foetal mouse visual cortex [16]. These exemplar studies indicate that the properties of neurons lacking FMRP are dependent of developmental stage and neuronal subtype being investigated. Indeed, in our system, intrinsic excitability and synaptic properties were unaltered. Furthermore, the lack of any differences in the passive and active membrane properties of FMRP-lacking human cortical neurons and control neurons contrasts with a previous report [21] that suggested ESC-derived neurons lacking FMRP were only capable of firing, at most, one action potential following a depolarizing current injection. While there are differences in the protocols used to generate pluripotent stem cell-derived neurons between these studies, our data do not support the notion that the human cortical neurons lacking FMRP generated using our culture protocols are hypoexcitable. Moreover, our data, while showing variability in the maximum number of action potentials produced following current injection, do not indicate that control and FMRP-lacking cortical neurons respond differently. Indeed, in all the parameters we have assessed, no significant differences were observed across any of the lines examined. Specifically, in regard to the maximum number of action potentials elicited by depolarizing current injections, while we do observe cells (irrespective of genotype) which only responded with a single action potential, the majority give rise to multiple action potentials during the depolarizing step. Analysis of synaptic function as assessed by measuring the properties of mEPSCs indicated that their frequencies and amplitude were not different between genotypes. This finding is in contrast to the studies on embryonic FXS stem cells that have reported decreased frequencies of spontaneous glutamatergic synaptic currents [20, 21].

FMRP has numerous target mRNAs that encode synaptically located proteins and more specifically that encode proteins associated with ion channels or their regulation [8]. Studies investigating prefrontal and entorhinal cortical neuron dysfunction in *Fmr1*^−/y^ mice concluded that the hyperexcitability displayed by these neurons was due to an increase in transient [38] and persistent [30] sodium currents. Our data show that in human neurons lacking FMRP, there is very little expression of the persistent sodium current and application of riluzole did not alter the profile of action potential bursting in these neurons. However, in control neurons, riluzole significantly reduced the duration of action potential bursts without affecting their overall frequency. This is in contrast to the observations seen in mouse models, whether this difference in the role of the persistent sodium current reflects a difference between human and mouse model systems or is a reflection of, for example, a more immature developmental stage of the human neurons used is not clear. Nevertheless, pharmacological activation of voltage-dependent sodium channels, by veratridine, in FMRP-lacking human cortical neurons resulted in a bursting profile that was very similar to that seen in control neurons. Moreover, veratridine had no effect on control neurons, possibly indicating that increasing voltage-dependent sodium channel activity in FMRP-null neurons can restore a bursting profile that is equivalent to that seen when FMRP is expressed.

FMRP modulates a number of potassium channels [8] and of specific interest for our present study is its regulation of large conductance calcium-activated potassium channels [45, 51, 52] where absence of FMRP results in lower current densities [31, 32, 46]. Indeed over-expression of the auxiliary β4 subunit has been shown to reduce cellular and circuit dysfunction [32], or activation of this conductance rescues sensory hypersensitivity and dendritic hyperexcitability [46] in *Fmr1*^−/y^ mice. In agreement with these rodent-based FXS models, we observed that human cortical neurons lacking FMRP had lower densities of large conductance calcium-activated potassium currents. Furthermore, blocking these channels with paxilline resulted in an alteration in the action potential bursting profile only in the control lines and was without effect in FMRP-null lines that suggests that the bursting profile seen in human FMRP-null neurons may also have an origin in dysregulated large conductance calcium-activated potassium channel expression.

In summary, our study demonstrates that in human cortical neurons, loss of FMRP results in an aberrant action potential bursting profile when compared to control neurons. This does not result from altered intrinsic and synaptic excitability but rather propose that this altered activity results from reduced Na_P_ and BK_Ca_ conductances. While reduced BK_Ca_ conductance has been reported in rodent models, it is important to highlight that our finding of reduced Na_P_ conductance contrasts with studies that have utilized rodent models.

## Limitations

Increased burst frequency has been reported in mouse *Fmr1*^−/y^ somatosensory cortical neurons [12] and *Fmr1*^−/y^ neurons in mouse cortical primary cultures [53]. However, the vast majority of neurons in our cultures are glutamatergic since there are no GABAergic interneurons, as assessed by staining for GAD [27–29]. As such, the properties of the network activity we recorded in our conditions are not equivalent to those recorded in rodent-based FXS models that have utilized either in vitro cultures, ex vivo slice preparations, or in vivo recording; such preparations will contain a variety of subpopulations of inhibitory interneurons [9, 10, 12–15, 30–32, 36, 37, 53]. Thus, direct comparisons of the type of network activity we have recorded are not necessarily prudent. However, it is interesting to note that a recent study using human iPSC-derived neurons and using extracellular multi-electrode array recordings reported an increase in spontaneous firing rate in FMRP-lacking neurons although no direct assessment of the profiles of action potential bursting was performed [23]. Notwithstanding these caveats, our study for the first time shows the robustness of the difference in the profile of activity observed in our control and FMRP-lacking human cortical neurons. This can be exploited to gain insight into the potential mechanistic basis for their distinct activity patterns. We also note that each of the lines display passive and active membrane properties (relatively depolarized resting membrane potentials, high input resistances, broad action potential half-widths and relatively low estimates of membrane capacitance) that are consistent with these neurons still being considered to be developmentally immature; again, this is consistent with our previous studies where we have investigated the physiological properties of human pluripotent stem cell-derived neurons [27–29, 54, 55].

## Conclusion

We conclude that pharmacological manipulations can alter the action potential burst profiles in both control and FMRP-null human cortical neurons, making them appear like their genetic counterpart. Our studies indicate that FMRP targets which have been found in rodent models of FXS are also potential targets in this human-based model system. Indeed, given the very distinct profiles of activity in the control and FMRP-null lines, it is advantageous to consider using an assessment of this activity as a surrogate for neuronal network activity in a human-based model system. Such studies complement research that use a variety of animal models, the latter having the advantage that many more types of investigation can be performed that cannot be carried out in a human-based system. Ultimately, the goal of our studies and those of others using pluripotent stem cell-derived neural tissue should be to gain better mechanistic insights into the dysfunction caused by loss of FMRP expression. This will hopefully allow new potential therapeutics to be assessed in an array of pre-clinical platforms that may result in greater translation to the clinic.

## Supplementary information

**Additional file 1: Figure S1.** Active and passive membrane properties of human pluripotent stem cell derived cortical neurons lacking FMRP are similar to the control neurons. (A) Representative current-clamp traces showing action potential firing in response to two depolarizing current injections (500 ms; +15 pA or +30 pA) from either *FMR1*^*+/y*^ (black trace) or *FMR1*^*/-y*^ (red trace) neurons. (B, C, D, E) Quantification of the passive membrane properties of hESC (*FMR1*^*+/y*^, *FMR1*^*-/y*^*)* and hiPSC (CON1, CON2, FXS1, FXS2, FXS3) derived cortical neurons illustrating no significant differences between control neurons and neurons lacking FMRP in their resting membrane potential (B), input resistance (C), capacitance (D) or rheobase current (E). (F, G, H, I) Quantification of action potential parameters in each of the lines indicating no difference in action potential (AP) threshold (F), AP amplitude (G), AP half-width (H) or the maximum number of APs fired in response to depolarizing current injections (I). One-way ANOVA with *post hoc* Tukey’s test. *FMR1*^*+/y*^: n = 19, N = 3; *FMR1*^*-/y*^: n = 15, N = 3; CON1: n = 25, N = 3; CON2: n = 18, N = 3; FXS1: n = 19, N = 3; FXS2: n = 33, N = 3; FXS3: n = 21, N = 3.

**Additional file 2: Figure S2.** Synaptic activity of human cortical neurons lacking FMRP is comparable to the control neurons. **(A)** Representative voltage-clamp traces of mEPSCs (V_hold_ = –70 mV) from either *FMR1*^*+/y*^ (black) and *FMR1*^*-/y*^ (red) neurons and recorded in the *presence* of TTX (500 nM). **(B)** As in (A) but from CON1 (black) or FXS1 (red) neurons. **(C)** Quantification of mEPSC frequencies indicating that these do not differ between each of the lines examined and had mean values of 0.443 ± 0.10 Hz (*FMR1*^*+/y*^; n = 10, N = 2); 0.403 ± 0.11 Hz (*FMR1*^*-/y*^; n =10, N = 2); 0.96 ± 0.18 Hz (CON1; n =23, N =3) and 0.78 ± 0.11 Hz (FXS1, n = 20, N = 3). **(D)** Quantification of mEPSC amplitudes indicating that these do not differ between each of the lines examined and had mean values of 20.85 ± 3.13 pA (*FMR1*^*+/y*^); 20.72 ± 3.08 pA (*FMR1*^*-/y*^); 26.3 ± 2.53 pA (CON1) and 29.92 ± 3.54 pA (FXS1). One-way ANOVA with post hoc Tukey’s test.

## Data Availability

The datasets used and/or analysed during the current study are available from the corresponding author on reasonable request.

## Abbreviations

AMPA: α-Amino-3-hydroxy-5-methyl-4-isoxazolepropionic acid
ANOVA: Analysis of variance
DAPI: 4′,6-Diamidino-2-phenylindole
FMRP: Fragile X mental retardation protein
FXS: Fragile X syndrome
GFAP: Glial fibrillary astrocyte protein
hESC: Human embryonic stem cell
hiPSC: Human induced pluripotent stem cell
hNA: Human nuclear antigen
NMDA: *N*-Methyl-d-aspartate
NPC: Neural progenitor cells
Oct 3/4: Octamer-binding transcription factor 4
SEM: Standard error of the mean
TTX: Tetrodotoxin
