# Supplementary figures and images for: Cortical neurons derived from human pluripotent stem cells lacking FMRP display altered spontaneous firing patterns
Source: Mol Autism. 2020 Jun 19;11:52. doi: 10.1186/s13229-020-00351-4 (PMC7304215; doi:10.1186/s13229-020-00351-4)

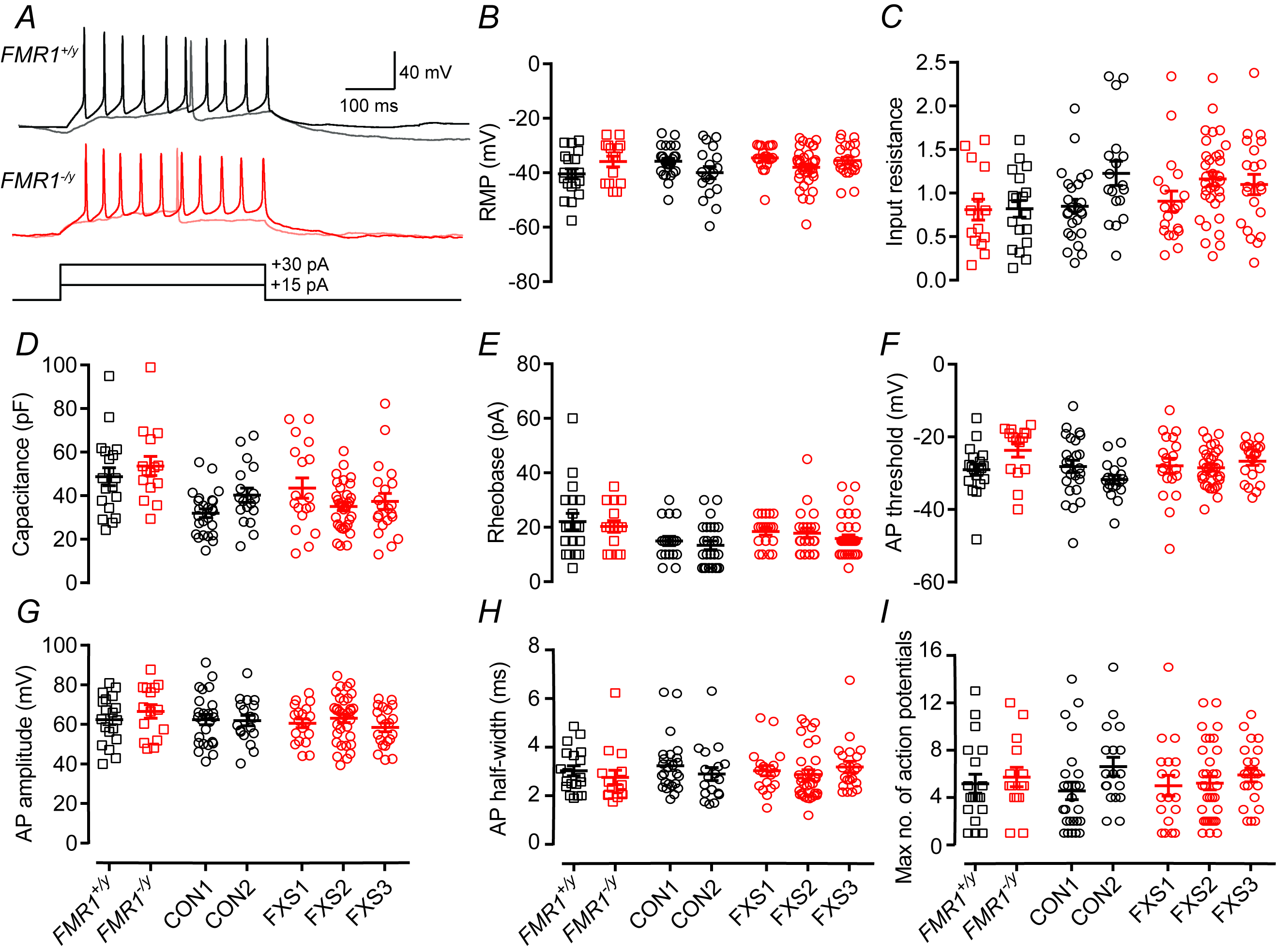

Supplement: Supplementary file 1 — Additional file 1: Figure S1. Active and passive membrane properties of human pluripotent stem cell derived cortical neurons lacking FMRP are similar to the control neurons. (A) Representative current-clamp traces showing action potential firing in response to two depolarizing current injections (500 ms; +15 pA or +30 pA) from either FMR1+/y (black trace) or FMR1/-y (red trace) neurons. (B, C, D, E) Quantification of the passive membrane properties of hESC (FMR1+/y, FMR1-/y) and hiPSC (CON1, CON2, FXS1, FXS2, FXS3) derived cortical neurons illustrating no significant differences between control neurons and neurons lacking FMRP in their resting membrane potential (B), input resistance (C), capacitance (D) or rheobase current (E). (F, G, H, I) Quantification of action potential parameters in each of the lines indicating no difference in action potential (AP) threshold (F), AP amplitude (G), AP half-width (H) or the maximum number of APs fired in response to depolarizing current injections (I). One-way ANOVA with post hoc Tukey’s test. FMR1+/y: n = 19, N = 3; FMR1-/y: n = 15, N = 3; CON1: n = 25, N = 3; CON2: n = 18, N = 3; FXS1: n = 19, N = 3; FXS2: n = 33, N = 3; FXS3: n = 21, N = 3. [file 13229_2020_351_MOESM1_ESM.tif]

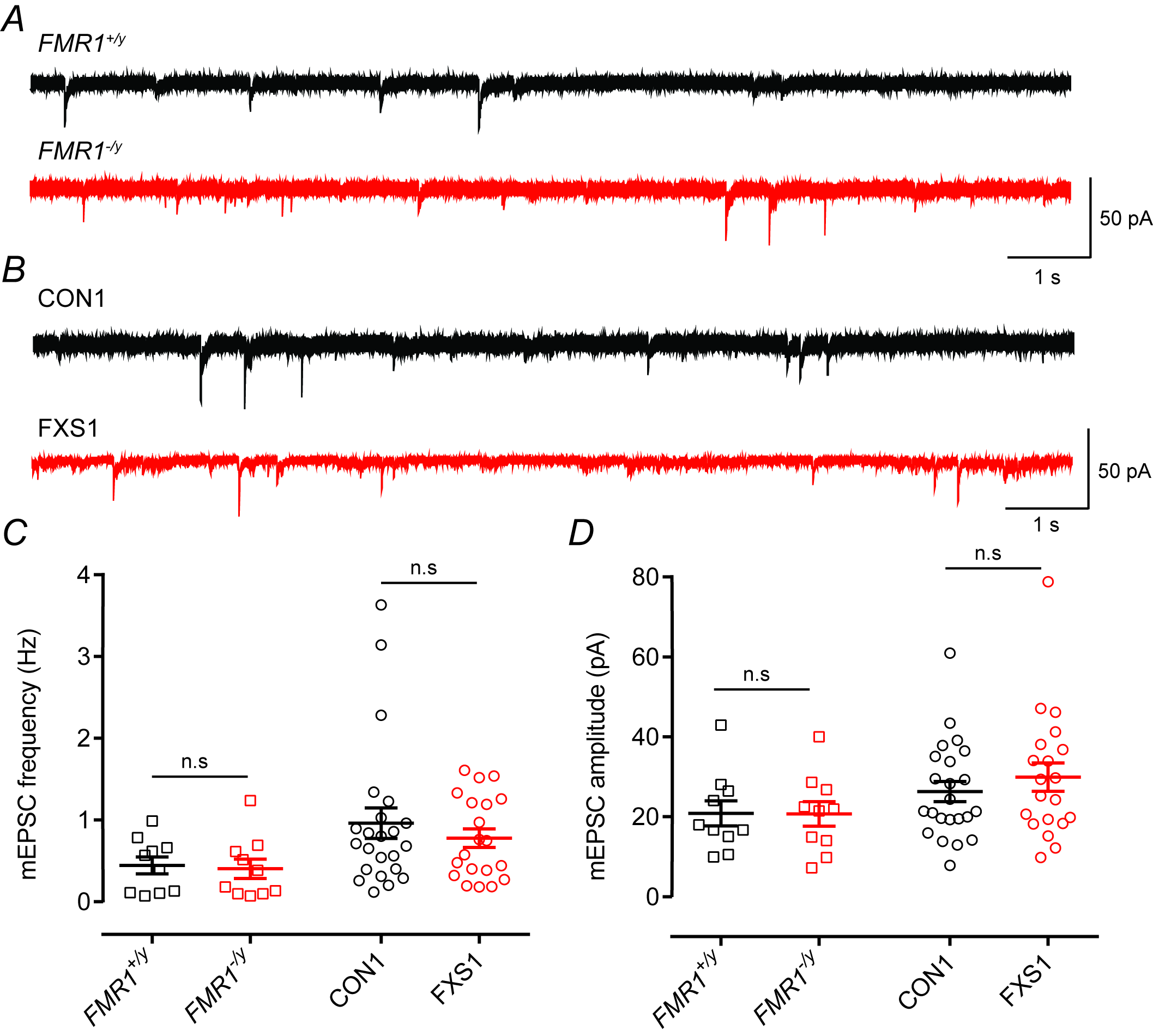

Supplement: Supplementary file 2 — Additional file 2: Figure S2. Synaptic activity of human cortical neurons lacking FMRP is comparable to the control neurons. (A) Representative voltage-clamp traces of mEPSCs (Vhold = –70 mV) from either FMR1+/y (black) and FMR1-/y (red) neurons and recorded in the presence of TTX (500 nM). (B) As in (A) but from CON1 (black) or FXS1 (red) neurons. (C) Quantification of mEPSC frequencies indicating that these do not differ between each of the lines examined and had mean values of 0.443 ± 0.10 Hz (FMR1+/y; n = 10, N = 2); 0.403 ± 0.11 Hz (FMR1-/y; n =10, N = 2); 0.96 ± 0.18 Hz (CON1; n =23, N =3) and 0.78 ± 0.11 Hz (FXS1, n = 20, N = 3). (D) Quantification of mEPSC amplitudes indicating that these do not differ between each of the lines examined and had mean values of 20.85 ± 3.13 pA (FMR1+/y); 20.72 ± 3.08 pA (FMR1-/y); 26.3 ± 2.53 pA (CON1) and 29.92 ± 3.54 pA (FXS1). One-way ANOVA with post hoc Tukey’s test. [file 13229_2020_351_MOESM2_ESM.tif]
